# Protective threshold of a potent neutralizing Zika virus monoclonal antibody in rhesus macaques

**DOI:** 10.1128/jvi.01429-24

**Published:** 2024-11-15

**Authors:** Joseph P. Nkolola, David Hope, Ruoran Guan, Alessandro Colarusso, Malika Aid, Deborah Weiss, John Misamore, Hanne Andersen, Mark G. Lewis, Lauren Williamson, Robert H. Carnahan, James E. Crowe, Dan H. Barouch

**Affiliations:** 1 Center for Virology and Vaccine Research, Beth Israel Deaconess Medical Center, Boston, Massachusetts, USA; 2 Bioqual, Rockville, Maryland, USA; 3 Vanderbilt Vaccine Center, Vanderbilt University Medical Center, Nashville, Tennessee, USA; 4 Department of Pediatrics, Vanderbilt University Medical Center, Nashville, Tennessee, USA; 5 Department of Pathology, Microbiology, and Immunology, Vanderbilt University Medical Center, Nashville, Tennessee, USA; The Ohio State University, Columbus, Ohio, USA

**Keywords:** Zika, biotherapeutic, antibody, non-human primate, efficacy

## Abstract

Zika virus (ZIKV) is a mosquito-borne flavivirus that caused a global pandemic in 2016–2017 with continued ongoing transmission at low levels in several countries. In the absence of an approved ZIKV vaccine, neutralizing monoclonal antibodies (mAbs) provide an option for the prevention and treatment of ZIKV infection. Previous studies identified a potent neutralizing human mAb ZIKV-117 that reduced fetal infection and death in mice following ZIKV challenge. In this study, we report exquisite potency of ZIKV-117-LALA-YTE, which has been engineered to reduce Fc receptor binding and to extend half-life, in a titration study in rhesus macaques to protect against ZIKV challenge. We show complete protection at a dose of 0.016 mg/kg ZIKV-117-LALA-YTE, which resulted in median serum concentrations of 0.13 µg/mL. The high potency of this mAb supports its potential clinical development as a novel biotherapeutic intervention for ZIKV.

**IMPORTANCE:**

In this study, we report the potency of the Zika virus (ZIKV)-specific neutralizing antibody ZIKV-117-LALA-YTE against ZIKV challenge in a titration study rhesus macaques. This high potency supports the further development of this monoclonal antibody for ZIKV.

## INTRODUCTION

Zika virus (ZIKV) is a member of the Flaviviridae family of positive-stranded RNA viruses and was first isolated in a rhesus macaque in Uganda in 1947 and in humans in 1952 (1, 2). Although primarily transmitted by the *Aedes aegypti* mosquito (3), human-to-human transmission can occur via sexual (4), vertical (5), and blood transfusion (6) routes. ZIKV outbreaks were reported in Micronesia in 2007 (7), Oceania in 2013–2014 (8), Brazil in 2015–2017 (9), and many countries in the Americas in 2016–2017 (10) and were declared a public health emergency of international concern by the World Health Organization (11). While 80% of persons infected with ZIKV are asymptomatic or mildly symptomatic, during the 2015–2017 Brazilian epidemic a causal link was established between ZIKV infection and microcephaly and other congenital malformations in pregnant women (12), as well as the neurological condition of Guillain–Barré syndrome in adults (13).

Although Zika cases have decreased significantly in frequency since their peak in 2017, the possibility of future outbreaks underscores the need for better preparedness, including the development of vaccines and biotherapeutics (14). A previous study reported the identification of a potent neutralizing human monoclonal antibody (mAb) ZIKV-117, which was isolated from an otherwise healthy individual with a history of symptomatic ZIKV infection (15). ZIKV-117 neutralized ZIKV strains belonging to African, Asian, and American lineages and mediated reduction of tissue pathology, placental and fetal infection, and mortality in murine models of experimental infection (15). More recently, a nanostructured lipid carrier delivering an alphavirus replicon encoding ZIKV-117 showed robust protection both as pre-exposure prophylaxis and post-exposure therapy in mice (16). The mechanism of neutralization afforded by the ZIKV-117 mAb involves binding to domain II of the E protein on the ZIKV surface and cross-linking E glycoprotein dimers, resulting in the prevention of the rearrangement of E proteins necessary for low-pH mediated fusion (17). In the current study, we assessed the potency of ZIKV-117-LALA-YTE, which includes LALA and YTE modifications that reduce Fc binding and extend half-life, in rhesus macaques to protect against ZIKV challenge.

## MATERIALS AND METHODS

### Study design

Twenty-four rhesus macaques, male (*n* = 12) and female (*n* = 12) animals ages 5–14 years, were housed at Bioqual, Rockville, MD, and the study was conducted in compliance with all relevant local, state, and federal regulations and was approved by the Bioqual Institutional Animal Care and Use Committee (IACUC). Six groups of rhesus macaques (*Macaca mulatta*) were intravenously (I.V.) infused on day −1 with ZIKV-117-LALA-YTE at doses of 2.0, 0.4, 0.08, 0.016, 0.0032, or 0 mg/kg. To assess protective efficacy against ZIKV challenge, all groups were challenged subcutaneously (S.Q.) on day 0 with 106 viral particles (VPs) (103 plaque-forming units) of ZIKV strain ZIKV-BR isolated from northeast Brazil (18, 19).

### Pharmacokinetics

Serum levels of the ZIKV-117-LALA-YTE were monitored using a previously described human IgG-specific enzyme-linked immunosorbent assay (ELISA) (20). In brief, ELISA plates were coated overnight at 4°C with 1 µg/mL of goat anti-human IgG (H + L) secondary antibody (monkey pre-adsorbed) (Novus Biologicals) and then blocked for 2 h. Serum samples were assayed at threefold dilutions starting at a 1:3 dilution in Blocker Casein in phosphate buffered saline (PBS) (Thermo Fisher Scientific) diluent. Samples were incubated for 1 h at ambient temperature and then removed, and plates were washed. Wells then were incubated for 1 h with horseradish peroxidase-conjugated goat anti-human IgG (monkey pre-adsorbed) (Southern Biotech) at a 1:4,000 dilution. Wells were washed and then incubated with SureBlue Reserve TMB Microwell Peroxidase Substrate (Seracare) (100 µL/well) for 3 min followed by TMB Stop Solution (Seracare) to stop the reaction (100 µL/well). Microplates were read at 450 nm. The concentrations of the human mAbs were interpolated from the linear range of concurrently run purified human IgG (Sigma) standard curves using Prism software, version 11.0 (GraphPad).

### RT-PCR

Plasma viral loads after ZIKV-BR challenge were monitored for 3 weeks using a previously established reverse transcription-polymerase chain reaction (RT-PCR) assay (19). In brief, the wild-type ZIKV BeH815744 Cap gene was used as a standard and was cloned into pcDNA3.1+, and the AmpliCap-Max T7 High Yield Message Maker Kit was used to transcribe RNA (Cellscript, WI, USA). RNA was purified using the RNA clean and concentrator kit (Zymo Research, CA, USA). Tenfold dilutions of the RNA standard were reverse transcribed and included with each RT-PCR assay. Viral loads were calculated as viral copies per milliliter. Assay sensitivity was 50 copies/mL.

### Statistics and modeling

A comparison of peak median ZIKV viral loads with sham controls was performed in Prism software (GraphPad, version 10.3.1) using adjusted Kruskal–Wallis tests. Estimation of a protective threshold for ZIKV-117-LALA-YTE prophylaxis was performed by comparing ZIKV-117-LALA-YTE concentration in serum at the time of challenge with the time-weighted average (TWA) values for the change of viral load in serum from day 0 to 10 after viral challenge as previously described (21). In brief, to compute TWA values, the area under the curve for the change in viral loads was calculated and divided by 10. TWA calculations were performed with the area under curve function in XY Analyses in GraphPad Prism (version 10.3.1). The TWA threshold was set up to <0.212 for full protection, ≥0.212 and ≤0.87 for partial protection, and >0.87 for no protection. The TWA value of 0.212 was imputed from a locally weighted scatterplot smoothing (LOWESS) fit applied to our data. More specifically, a TWA of 0.212 is the associated dependent value to the minimum antibody concentration measured on the day of challenge in fully protected animals. The curve was estimated in R (version 4.3.1) using the LOWESS function from the stats package, with default parameters. Visualization of the TWA and the fitted LOWESS curve was done in ggplot2 (version 3.5.1).

## RESULTS AND DISCUSSION

**FIG 1:**
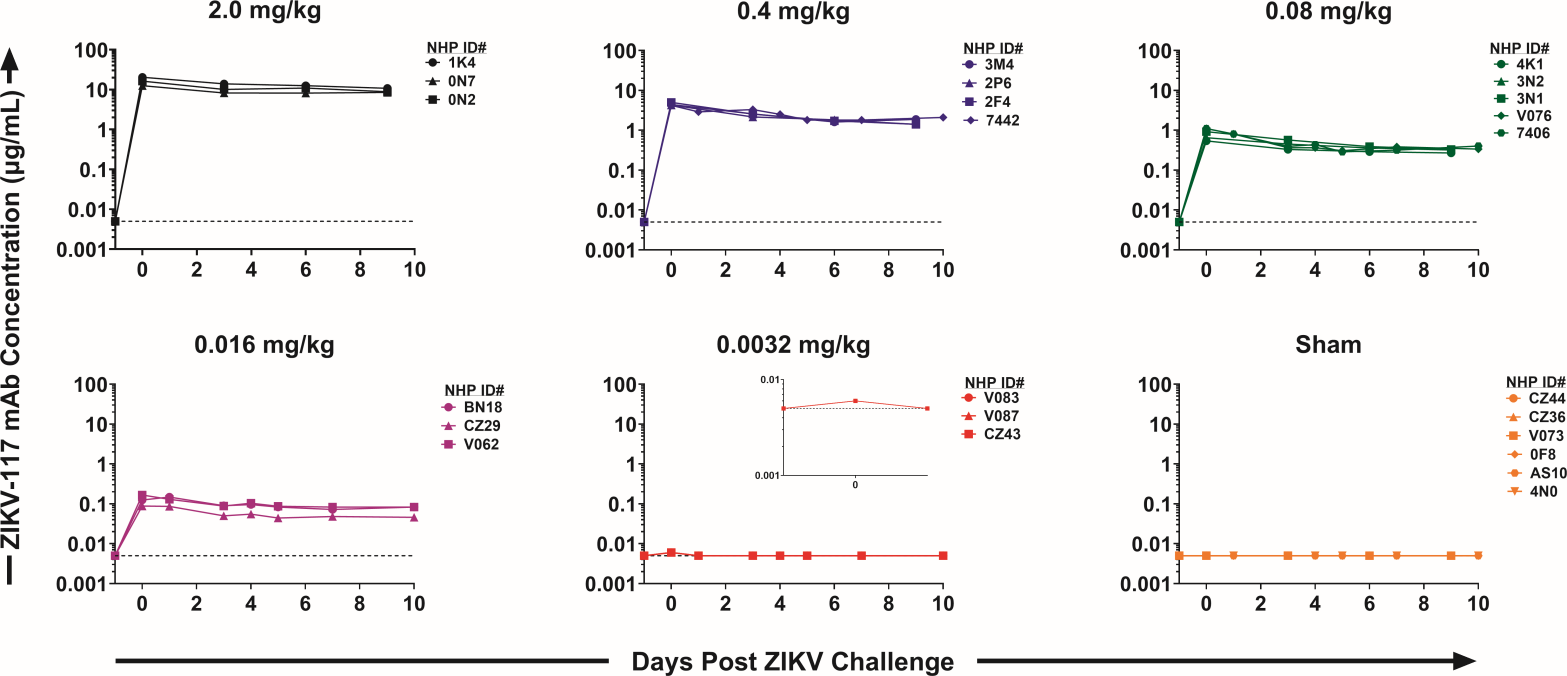
ZIKV-117-LALA-YTE pharmacokinetics following infusion as measured by a human IgG-specific enzyme-linked immunosorbent assay (ELISA). Each line correspondences to a single animal. The horizontal dashed line represents the assay limit of detection (0.005 µg/mL).

Twenty-four rhesus macaques received an I.V. infusion of 2.0 (*N* = 3), 0.4 (*N* = 4), 0.08 (*N* = 5), 0.016 (*N* = 3), 0.0032 (*N* = 3), or 0 (*N* = 6) mg/kg ZIKV-117-LALA-YTE on day −1. Serum ZIKV-117 levels were determined through day 10 by ELISA and showed the expected biphasic profile involving a rapid distribution phase followed by a slower elimination phase (Fig. 1). Peak mAb levels were observed on day 0, 1 day following mAb administration. Animals that received 2.0, 0.4, 0.08, 0.016, 0.0032, and 0 mg/kg ZIKV-117 exhibited mean peak concentrations of 16.4, 4.5, 0.9, 0.13, 0.006, and 0 µg/mL, respectively, on day 0 (Fig. 1). Circulating ZIKV-117-LALA-YTE levels were observed over 10 days in all groups that received ZIKV-117-LALA-YTE, except for the 0.0032 mg/kg group for which we only detected borderline ZIKV-117-LALA-YTE levels on day 0. No ZIKV-117-LALA-YTE levels were detected in the sham control group.

**FIG 2:**
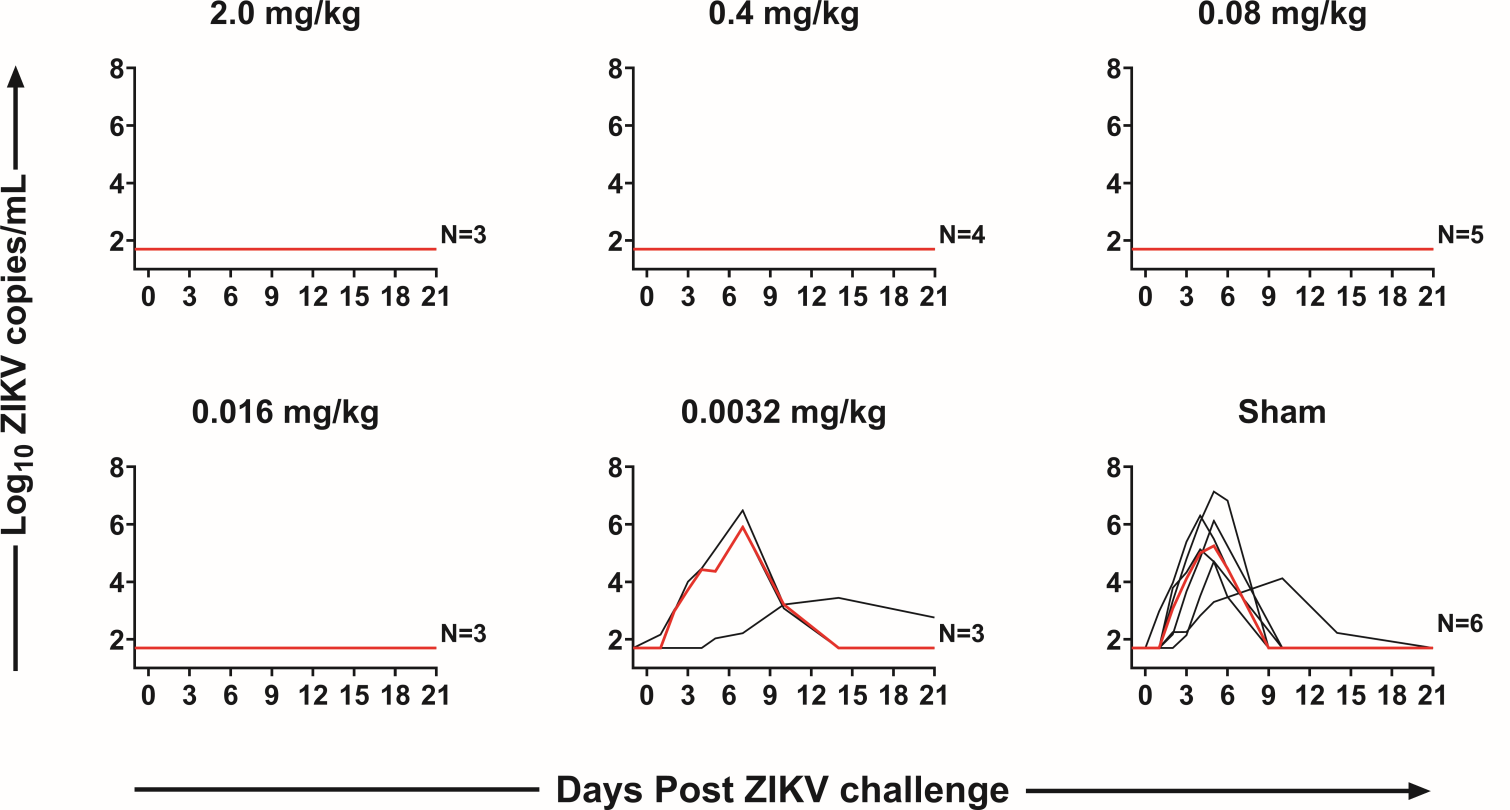
ZIKV-BR viral loads following challenge as determined by reverse transcription-PCR (RT-PCR). Each black line corresponds to a single animal, and the red line depicts the median for each group.

**FIG 3:**
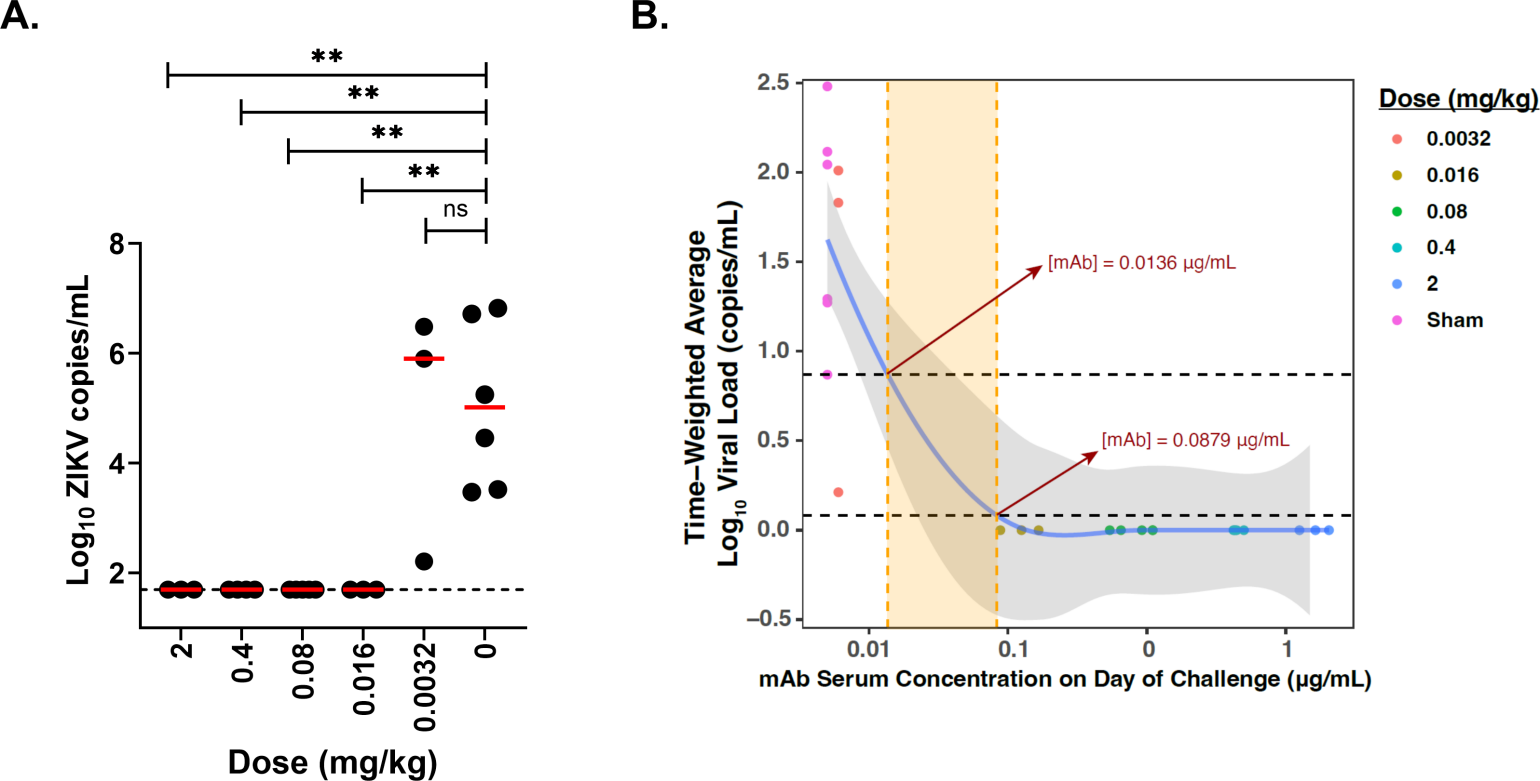
(**A**) Comparison of peak median Zika virus (ZIKV) viral loads in each group compared with sham controls. The horizontal dashed line represents the assay limit of detection (50 copies/mL). Adjusted Kruskal–Wallis tests are shown; **P < 0.05. (**B**) Time-weighted average (TWA) values for the change of viral loads from day 1 to 10 after viral challenge (y-axis) compared with log serum ZIKV-117-LALA-YTE concentrations on the day of challenge (x-axis). The fitting curve was estimated using the locally weighted scatterplot smoothing (LOWESS) method and is shown in purple, and gray shading indicates the 95% CI. Horizontal black dotted lines indicate designated TWA thresholds for full (bottom line) and partial (top line) protection. Vertical dotted orange lines indicate maximal and minimal predicted cutoff for protective antibody concentration in serum.

All animals were challenged on day 0 with 106 VP ZIKV-BR by the S.Q. route. The ZIKV-BR strain has been reported to recapitulate key clinical indications in wild-type SJL mice, including fetal microcephaly and intrauterine growth restriction (18). Sham-inoculated animals demonstrated median peak viral loads of 4.7 log_10_ copies/mL (range 3.3–7.1 log_10_ copies/mL; *n* = 6) on days 5–6 following challenge (Fig. 2). In contrast, animals that received 2.0, 0.4, 0.08, and 0.016 mg/kg ZIKV-117-LALA-YTE showed complete protection with no detectable viremia (<50 copies/mL) at all time points (Fig. 2 and 3A). Animals that received 0.0032 mg/kg ZIKV-117-LALA-YTE showed median peak viral loads of 5.9 log_10_ copies/mL (range 2.2–6.5 log_10_ copies/mL; *n* = 3) on days 6–7 following challenge, comparable to the sham control animals (Fig. 2 and 3A).

We observed complete protection at a dose of 0.016 mg/kg ZIKV-117-LALA-YTE, which corresponded to median serum concentrations of 0.13 µg/mL (Fig. 2 and 3A). To model the threshold for protection more accurately, a fitting curve was estimated using the LOWESS method, which suggested that the ZIKV-117-LALA-YTE protective threshold was between 0.014 and 0.088 µg/mL (Fig. 3B). These data demonstrate the high potency of ZIKV-117-LALA-YTE for protection against ZIKV-BR challenge in rhesus macaques (Fig. 3B).

At least 89 countries and territories have had mosquito-borne transmission of ZIKV (22). In this study, we show that ZIKV-117-LALA-YTE is exquisitely potent for ZIKV protection in rhesus macaques. Doses of 0.016 mg/kg ZIKV-117-LALA-YTE provided complete protection in this model, suggesting that 0.13 µg/mL is above the minimum protective threshold for this antibody. While several potently neutralizing antibodies that target the conformational epitope of surface E glycoprotein dimers of both dengue virus and ZIKV have been identified (23), ZIKV-117 remains the only ultrapotent ZIKA-specific neutralizing mAb to the best of our knowledge (24). Taken together, our data suggests the potential of ZIKV-117-LALA-YTE as a novel biotherapeutic for ZIKV.

## Data Availability

The data that support the findings of this study are available from the corresponding author upon request.
